# Fresh *versus* elective frozen embryo transfer: Cumulative live birth rates of 7,236 IVF cycles

**DOI:** 10.5935/1518-0557.20210094

**Published:** 2022

**Authors:** Thi Minh Chau Le, Phuc Thinh Ong, Quoc Anh Nguyen, Matheus Roque

**Affiliations:** 1 Department of Infertility, Tu Du Hospital, Ho Chi Minh City, Viet Nam, Postal Code 70000; 2 Center for Population Health Sciences, Hanoi University of Public Health, Viet Nam, Postal Code 70000; 3 Department of Reproductive Medicine, MATER PRIME, São Paulo, Brazil, Postal Code 04029-200

**Keywords:** freeze-all, elective frozen-thawed embryo transfer, fresh embryo transfer, cumulative live birth rate, cryopreservation

## Abstract

**Objective:**

To determine whether elective frozen embryo transfer (eFET), or the 'freeze-all' strategy, associated with better cumulative clinical outcomes compared with fresh embryo transfer (ET).

**Methods:**

A total of 7,236 IVF cycles that were followed by a fresh ET or eFET between 2013 and 2017. The patients were subjected to controlled ovarian stimulation (COS) with a gonadotropin-releasing hormone (GnRH) antagonist protocol and cleavage-stage ET. Embryo cryopreservation was performed on day 3 by vitrification using an open system. A comparison of cumulative outcomes between the eFET (n=4,065cycles) and the fresh ET groups (n=3,171cycles) were performed. The analysis was performed in four groups of patients based on the number of retrieved oocytes: Group 1: poor responders (1-3 oocytes); Group 2: suboptimal responders (4-9 oocytes); Group 3: normal responders (10-15 oocytes); and Group 4: hyper-responders (>15 oocytes). The primary outcome was the cumulative live birth rate (CLBR) per stimulated cycle.

**Results:**

There were a total of 10,283 ETs (n=5,639 eFET group; n=4,644 fresh group). The freeze-all strategy is associated with improved CLBRs in normal and hyper-responders, but not in suboptimal and poor responders. In Group 1, there were 351 IVF cycles and 387 ETs in total, and the CLBR was 14.3% and 17.7% (p=0.584) for the eFET and fresh group, respectively. In Group 2, there were 2,074 IVF cycles and 2,465 ET in total, and the CLBR was 25.1% and 23.3% (*p*=0.083) in the eFET and fresh group, respectively. There was a significant difference in the CLBR in Groups 3 and 4, favouring the eFET strategy. In Group 3, 2226 IVF cycles and 3243 ET were performed. The CLBR was 40.5% in the eFET and 36.6% in the fresh group (*p*<0.001). In Group 4, there were 2547 IVF cycles and 3,188 ET in total, and the CLBR was 52.2% and 47.7% (*p*<0.001) in the eFET and fresh group, respectively. The number needed to treat to achieve one additional live birth was 25.9 in Group 3 and 22.3 in Group 4.

**Conclusions:**

The implementation of the freeze-all strategy should be individualized. The freeze-all strategy is associated with improved CLBRs in normal and hyper-responders, but not in suboptimal and poor responders.

## INTRODUCTION

Despite the substantial advancements in in vitro fertilization (IVF), experts in the field continue to search for the most effective protocols for maximizing patient outcomes. One of the most important advances in assisted reproductive technology (ART) in recent years is associated with improvements in cryopreservation techniques, which have fundamentally transformed the way we perform IVF. The advent and improvement in vitrification protocols has led to high rates of embryo survival after the thawing process, and at least the same clinical results for frozen-thawed embryo transfer (FET) as for fresh embryo transfer (ET) (Nagy *et al*., 2020). This advance in cryopreservation protocols is associated with improvements in the cumulative live birth rate (CLBR) per cycle, and also the implementation of a so-called freeze-all strategy during an IVF cycle. With this strategy, fresh ET is not performed, and all viable embryos are electively cryopreserved (Shapiro *et al*., 2014; Roque *et al*., 2018a). These improvements have changed daily IVF practice, as can be observed by evaluating the number of IVF cycles performed for freeze-all embryos and/or oocytes in United Stated. Over just ten years (from 2007 to 2016), there was a 33-fold increase in the number of freeze-all cycles, from 2,020 freeze-all cycles performed in 2007 to 65,840 performed in 2016 (Nagy *et al*., 2020). However, it is still not unequivocal for which patients this strategy should be implemented (Roque & Esteves, 2020).

Fresh ET, in which the best morphological-quality embryo(s) is/are transferred in a stimulated cycle and all surplus viable embryos with adequate quality are frozen for the future use, is a routine practice in IVF cycles. However, according to this strategy, the risk of ovarian hyperstimulation syndrome (OHSS) increases with the increase in ovarian response to controlled ovarian stimulation (COS) (Steward *et al*., 2014). In addition, there is evidence of an association between COS and adverse effects on the endometrium, and subsequently on endometrial receptivity. The supra-physiologic hormonal levels that occur during a COS may be associated with modifications in the peri-implantation endometrium that may be related to a decrease in pregnancy rates in fresh ET compared with FET. These modifications are related to an endometrial advancement that can be observed during histological evaluation during a fresh cycle, and when this advancement occurs over 3 days, no pregnancies are achieved (Ubaldi *et al*., 1997; Kolibianakis *et al*., 2002). There are also changes in gene expression profiles in the endometrium of patients subjected to COS, suggesting that ovarian hyperstimulation and high progesterone levels on the day of final oocyte maturation may be detrimental to implantation by altering gene expression crucial for endometrium-embryo interaction (Horcajadas *et al*., 2005; Labarta *et al*., 2011).

The freeze-all strategy has been suggested as a suitable alternative to fresh transfer in order to reduce OHSS and overcome negative effects on the endometrium during COS, and to enhance the clinical outcome of the IVF cycles (Roque *et al*., 2017a; Blockeel *et al*., 2016). However, eFET requires a lab with specific expertise in freezing protocols, and may be associated with an increase in the laboratory workflow, cost of treatment, and also with a delay in achieving the pregnancy (Blockeel *et al*., 2019). Importantly, the evidence of benefit from a freeze-all policy for patients is not unequivocal (Roque *et al*., 2018a; Wong *et al*., 2017; Li *et al*., 2019). It is uncertain for which group of patients this strategy is most beneficial. Different results from different studies have been reported. Better results for high responders using elective frozen embryo transfer (eFET) have been reported by some authors (Chen *et al*., 2016; Shapiro *et al*., 2011a) but not by others (Li *et al*., 2019). Similarly, some authors have found that this strategy may be associated with better IVF outcomes in normal responders (Roque *et al*., 2017b; Shapiro *et al*., 2011b), whereas others did not (Shi *et al*., 2018; Vuong *et al*., 2018). Reports for poor responders have been scarce (Roque *et al*., 2018a). Interestingly, most studies compared the fresh to the freeze-all strategy only in the first ET, and did not evaluate the strategy in different groups of patients based on ovarian response during COS.

In this study, we aim to evaluate whether the freeze-all strategy is associated with better cumulative clinical outcomes per cycle than with fresh ET in subgroups of patients from poor to high ovarian response, and to identify which subgroups may benefit from this strategy.

## MATERIALS AND METHODS

A retrospective cohort study was conducted between January 2013 and December 2017 in the IVF unit of Tu Du hospital, Vietnam. The study was approved by an institutional review board.

### Patient selection

The patients enrolled in this study fulfilled the following inclusion criteria: 1) cleavage-stage ET (day 3); 2) gonadotropin-releasing hormone (GnRH) antagonist protocols; 3) female subjects aged 18 to 45 years old. The exclusion criteria were as follows: 1) oocyte donor cycles; 2) cycles with fresh ET after a GnRH agonist trigger; 3) surrogacy treatments; 4) previous recurrent miscarriage; 5) implantation failure (≥3 previous ETs without pregnancy); 6) severe male factor infertility (oligospermia <1 million/mL and azoospermia); 7) uterine pathology; 8) those on cycles with preimplantation genetic testing (PGT); 9) in vitro maturation (IVM) cycles.

### Treatment protocol

COS was performed with either recombinant follicle-stimulating hormone (rFSH) or highly purified human menopausal gonadotropin (hp-hMG), with a starting dose ranging from 100 IU to 450 IU per day based on the patient's age and ovarian reserve tests. The gonadotropin dose was adjusted based on the ovarian response and hormonal parameters. A fixed GnRH antagonist protocol was used starting on day 5 of the stimulation cycle. Cycle monitoring was performed through transvaginal ultrasound scans and hormonal measurements (estradiol, LH and progesterone). Final oocyte maturation was triggered with human chorionic gonadotropin (hCG) when at least three follicles of 18 mm diameter were observed, or triptorelin if there were more than 20 follicles over 11 mm on the trigger day.

Oocyte retrieval was performed under transvaginal ultra-sound guidance after 36-38 hours of the trigger, followed by intracytoplasmic sperm injection (ICSI). The fertilized oocytes were then cultured up to day 3 after oocyte pick up. The embryos were graded according to their cell number, blastomere regularity, and fragmentation degree (Alpha Scientists in Reproductive Medicine and ESHRE Special Interest Group of Embryology, 2011), and the ET was performed (fresh group) and surplus embryos were cryopreserved when available. For fresh ET, the luteal phase support started on the day of oocyte retrieval with 600 mg vaginal micronized progesterone daily, until the 9^th^ week of pregnancy, when pregnancy occurred. The freeze-all group had all viable embryos cryopreserved on the 3^rd^ day of embryo development, and the strategy was implemented when: 1. serum progesterone was >1.5 ng/mL on the trigger day; 2. endometrial thickness <7 mm on the trigger day; 3. more than 20 oocytes retrieved; 4. based on patient's request.

### Cryopreservation/thawing and endometrial preparation

In case of freeze-all and for the surplus embryos in the fresh group, the embryos were vitrified on day 3 using an open system as previously described (Kuwayama *et al*., 2005). First, the embryos were exposed to the equilibrium solution. Then, they were exposed to the vitrification solution for 30 s. Afterwards, the embryos were placed on top of the strip with a very small amount of vitrification solution, and the strips were then immersed into liquid nitrogen. Sheaths were put on to cover the strips with vitrified embryos, and the embryos were kept in liquid nitrogen tanks.

When thawing, the strips were immersed into thawing solution at 37°C for 60s immediately after being removed from the plastic sheaths. Afterward, the thawed embryos were put into a dilution solution for 3 min at room temperature, and a buffer solution was then used to wash the embryos twice for 10 min in total. After being thawed, the embryos were assessed according to morphological criterion, and they were considered viable if more than 50% of the cells were intact.

An FET cycle was started with endometrial priming on the second day of the menstrual cycle using 6 to 8 mg/day of estradiol valerate orally. Estradiol priming was used for about 14 to 20 days, and an ultrasound was performed to evaluate the endometrium thickness. If the endometrium thickness was ≥7mm, the FET was scheduled, and vaginal micronized progesterone was started 3 days prior to the ET. Progesterone was used until 9 weeks of pregnancy, while estradiol valerate was used until a foetal heartbeat was confirmed.

### Outcomes and subgroups of patients evaluated

The main outcome was the CLBR per oocyte retrieval, defined as the number of deliveries with at least one live birth resulting from one aspirated ART cycle, including all cycles in which fresh and/or frozen embryos were transferred, until one delivery with a live birth occurred or until all embryos were used, whichever occurred first (24), following a fresh or eFET strategy. The complete expulsion or extraction from a woman of a product of fertilization, after 22 completed weeks of gestational age (ICMART).

The secondary outcomes were pregnancy rate, clinical pregnancy rate, implantation rate, miscarriage rate, and OHSS. Pregnancy was determined by hCG levels measured 11 days after ET. Clinical pregnancy was defined by observation of intrauterine embryo heart motion by 7 weeks of gestation. Ongoing pregnancy was defined as pregnancy proceeding beyond the 12^th^ week of gestation. Miscarriage was defined as a spontaneous loss of a clinical pregnancy before 22 completed weeks of gestational age. The implantation rate was calculated as the ratio of the number of observed embryo heartbeats to the number of transferred embryos. OHSS is known as "an exaggerated systemic response to ovarian stimulation characterized by a wide spectrum of clinical and laboratory manifestations. It may be classified as mild, moderate, or severe according to the degree of abdominal distention, ovarian enlargement, and respiratory, hemodynamic, and metabolic complications" (Zegers-Hochschild *et al*., 2017).

Cycles were classified according to strategy (fresh or freeze-all), and stratified into four groups by the number of oocytes retrieved, namely poor ovarian response (1-3 oocytes retrieved), suboptimal ovarian response (4-9), normal ovarian response (10-15 oocytes retrieved), and hyper ovarian response (>15 oocytes retrieved) (Polyzos & Sunkara, 2015; Drakopoulos *et al*., 2016).

### Statistical analysis

The continuous data are presented as mean value±standard deviation (SD), or median with interquartile range (IQR). The categorical data are described with frequency and percentage. The quantitative variables were analysed with the Student's t or Wilcoxon rank sum test as appropriate. For the comparison of categorical data, the Chi-squared test or Fisher's exact test was performed. Differences were considered significant when *p*<0.05.

Multivariable logistic regression was performed to control for potential confounders, including age, indications for IVF, endometrial thickness on trigger day, number of previous IVF attempts, duration of infertility, number of high-scoring embryos, average number of transferred embryos, number of embryos frozen, number of retrieved oocytes, duration of ovarian stimulation, and total dose of gonadotropin. The adjusted odds ratios (aOR) of CLBR with 95% CI between fresh and freeze-all strategies were reported for each group of oocytes retrieved. Statistical analyses were performed using R software version 3.6.1.

## RESULTS

A total of 7,236 ICSI cycles and 10,283 ETs were included in the study, of which 5,639 followed freeze-all strategy and 4,644 followed the fresh strategy (Table 1). The baseline and clinical characteristics of patients are shown in Table 2, categorized by group for number of oocytes retrieved. Within groups 1 to 3, no significant difference were found between freeze-all and fresh patients regarding type of infertility, the number of previous IVF attempts, indications for IVF, and fertilization rate. In Group 4, there was no statistically significant difference between freeze-all and fresh patients when age, duration of infertility, number of previous IVF attempts, duration of ovarian stimulation, and cleavage rate were evaluated.

**Table 1.** Numbers of cycles and numbers of embryo transfers.

Number of oocytes retrieved	Number of IVF/ICSI cycles		Number of embryos transferred	
	Fresh	Freeze-all	Fresh	Freeze-all
**1-3 (Group 1)**	232	119	265	122
**4-9 (Group 2)**	1086	988	1316	1149
**10-15 (Group 3)**	1090	1174	1662	1581
**>15 (Group 4)**	763	1784	1401	2787
**Total**	3171	4065	4644	5639
	7236		10283	

**Table 2.** Baseline and clinical characteristics of patients.

Characteristics	Group 1 (1-3 oocytes)			Group 2 (4-9 oocytes)			Group 3 (10-15 oocytes)			Group 4 (>15 oocytes)		
Age (years)	34.23±5.37	36.73±4.80	<0.001	33.1±5.02	34.18±4.75	<0.001	31.5±4.25	32.04±4.43	0.003	30.67±4.40	30.48±4.12	0.319
Duration of infertility (years)	5.3±3.67	6.56±4.14	0.006	5.67±3.99	6.06±3.97	0.029	5.24±3.39	5.73±3.66	<0.001	5.15±3.22	5.04±3.14	0.447
Type of infertility (n;%) Primary Secondary	157 (67.7) 75 (32.3)	75 (63.0) 44 (37.0)	0.383	727 (67.0) 358 (33.0)	663 (67.2) 324 (32.8)	0.935	760 (69.7) 330 (30.3)	852 (72.6) 322 (27.4)	0.135	534 (70.2) 227 (29.8)	1306 (73.2) 477 (26.8)	0.112
Previous IVF attempts (n)	1.19±0.93	1.20±0.65	0.927	1.13±0.41	1.15±0.76	0.505	1.15±0.51	1.13±0.68	0.559	1.13±0.42	1.14±0.85	0.915
Indications for IVF (n;%) Tubal factor Male factor Female factor Ovulation disorder Endometriosis Unexplained infertility Others	28 (12.1) 90 (39.0) 64 (27.7) 3 (1.3) 0 (0.0) 4 (1.7) 42 (18.2)	7 (5.9) 42 (35.3) 46 (38.7) 0 (0.0) 0 (0.0) 0 (0.0) 24 (20.2)	0.084**	153 (14.1) 514 (47.4) 203 (18.7) 8 (0.7) 6 (0.6) 9 (0.8) 191 (17.6)	126 (12.8) 426 (43.2) 211 (21.4) 6 (0.6) 4 (0.4) 6 (0.6) 206 (20.9)	0.228	184 (16.9) 588 (53.9) 90 (8.3) 8 (0.7) 5 (0.5) 9 (0.8) 206 (18.9)	173 (14.8) 634 (54.1) 82 (7.0) 21 (1.8) 3 (0.3) 6 (0.5) 252 (21.5)	0.085	97 (12.7) 453 (59.5) 41 (5.4) 11 (1.4) 4 (0.5) 4 (0.5) 151 (19.8)	241 (13.5) 1025 (57.5) 65 (3.6) 83 (4.7) 2 (0.1) 14 (0.8) 352 (19.8)	<0.001
Antral Follicle Count (n)	10.83±6.96	7.73±5.20	0.001	12.14±6.62	10.59±6.59	<0.001	17.18±8.00	15.66±8.18	0.001	19.66±8.17	21.78±9.74	<0.001
AMH (ng)	1.7 (1.1-2.9)	1.6 (1.0-2.5)	0.231*	2.3 (1.4-3.8)	2.4 (1.7-3.8)	0.02*	3.7 (2.5-5.2)	3.9 (2.6-5.8)	0.01*	4.8 (3.3-6.9)	6.3 (4.2-9.1)	<0.001*
Days of ovarian stimulation (n)	10.2±1.48	10.33±1.63	0.466	10.15±1.35	10.06±1.22	0.082	10.2±1.26	10.07±1.16	0.010	10.25±1.08	10.29±1.19	0.440
Total gonadotropin dose (IU)	2749.13± 765.5	2973.89± 678.46	0.006	2742.03± 743.62	2809.55± 657.56	0.031	2500.46± 808.11	2538.39± 799.4	0.273	2227.7± 823.94	2105.87± 844.82	<0.001
On hCG trigger day Estradiol level (pg/ml)	1796	1584	0.044*	2090	2000	0.217*	2873	2678	0.076*	3316	3956	<0.001*
	(1208-2782)	(1194-2133)		(1431-3253)	(1440-2960)		(1948-4068)	(1901-4032)		(2301-6638)	(2740-7787)	
Progesterone level (ng/ml)	0.7	0.8	0.608*	0.8	0.8	0.554*	0.9	0.9	0.014*	0.9	1.1	<0.001*
	(0.5-1.0)	(0.5-1.0)		(0.6-1.1)	(0.6-1.1)		(0.6-1.2)	(0.7-1.3)		(0.7-1.2)	(0.8-1.5)	
Endometrial thickness (mm)	10.83±1.5	10.41±1.06	0.003	10.78±1.35	10.35±1.03	<0.001	10.91±1.36	10.37±1.01	<0.001	10.93±1.31	10.33±0.98	<0.001
Oocytes retrieved (n)	2.44±0.72	2.52±0.64	0.254	6.61±1.71	6.87±1.64	<0.001	12.34±1.66	12.3±1.72	0.595	19.09±3.12	22.84±6.59	<0.001
Metaphase II oocytes (n)	1.93±0.78	2.26±0.67	<0.001	5.43±2.08	5.96±1.72	<0.001	10.36±2.47	10.4±2.35	0.739	15.22±3.66	19.18±5.85	<0.001
Fertilization Rate	0.79±0.25	0.82±0.26	0.316	0.71±0.23	0.73±0.21	0.050	0.70±0.20	0.71±0.18	0.504	0.70±0.18	0.73±0.16	0.012
Cleavage Rate	1.0 (1.0-1.0)	1.0 (1.0-1.0)	0.015	1.0 (0.8-1.0)	0.8 (0.7-1.0)	<0.001	0.8 (0.7-1.0)	0.8 (0.6-0.9)	0.003*	0.8 (0.6-0.9)	0.8 (0.6-0.9)	0.429*
High-score embryos on Day 3 (n)	1 (0 - 1)	0 (0 - 1)	0.310*	1 (0 - 2)	1 (0 - 2)	0.660*	2 (0 - 4)	1 (0 - 2)	<0.001*	3 (0 - 7)	2 (0 - 3)	<0.001*
Average embryos transferred (n)	1.71±0.77	1.78±0.70	0.442	2.58±0.79	2.53±0.70	0.074	2.88±0.57	2.71±0.57	<0.001	2.97±0.45	2.84±0.49	<0.001
Embryos frozen (n)	1.25±0.77	1.77±0.73	<0.001	3.44±1.55	3.47±1.49	0.705	5.76±2.23	5.62±2.33	0.145	8.2±3.29	10.52±4.78	<0.001

Table 3 presents the IVF outcomes for each group. An analysis of live birth rate (LBR) in the first ET of fresh and freeze-all cycles showed no significant difference in Group 1 (*p*=0.983). Interestingly, the freeze-all outperformed the fresh strategy in all other groups (*p*=0.004 in Group 2, *p*<0.001 in Group 3 and Group 4). Except for patients with 1-3 oocytes retrieved, the first cycle LBR for the freeze-all was always higher than the fresh policy.

**Table 3.** Comparison of IVF outcomes between freeze-all and fresh embryo transfer strategy.

IVF outcomes	Group 1 (1-3 oocytes)			Group 2 (4-9 oocytes)			Group 3 (10-15 oocytes)			Group 4 (>15 oocytes)		
	Fresh	Freeze-all	*p*	Fresh	Freeze-all	*p*	Fresh	Freeze-all	*p*	Fresh	Freeze-all	*p*
Live birth rate 1^st ^embryo transfer (ET) Singleton livebirth per women Twin livebirth per women Total live birth per women	24 (10.3) 7 (3.0) 31 (13.4)	12 (10.1) 4 (3.4) 16 (13.4)	0.939 1.000* 0.983	146 (13.4) 45 (4.1) 191 (17.6)	151 (15.3) 71 (7.2) 223 (22.6)	0.232 0.003 0.004	166 (15.2) 62 (5.7) 229 (21.0)	266 (22.7) 113 (9.6) 379 (32.3)	<0.001 <0.001 <0.001	111 (14.5) 43 (5.6) 154 (20.2)	411 (23.0) 236 (13.2) 648 (36.3)	<0.001 <0.001 <0.001
Pregnancy rate 1^st^ ET	37 (15.9)	27 (22.7)	0.122	260 (24.0)	316 (32.0)	<0.001	310 (28.4)	509 (43.4)	<0.001	215 (28.2)	853 (47.8)	<0.001
Clinical pregnancy rate 1^st^ ET	31 (13.4)	17 (14.3)	0.811	196 (18.0)	233 (23.6)	<0.002	241 (22.1)	388 (33.0%)	<0.001	160 (21.0)	670 (37.6)	<0.001
Implantation rate 1^st^ ET	0.09±0.24	0.1±0.26	0.612	0.09±0.21	0.14±0.27	<0.001	0.11±0.22	0.19±0.29	<0.001	0.1±0.21	0.22±0.3	<0.001
Miscarriage	0 (0.0)	1 (0.8)	0.315*	0 (0.0)	7 (0.6)	0.005*	7 (0.4)	15 (0.9)	0.067	6 (0.4)	29 (1.0)	0.040
Ectopic Pregnancy	2 (0.8)	0 (0.0)	1.000*	13 (1.0)	8 (0.7)	0.432	17 (1.0)	20 (1.3)	0.516	20 (1.4)	30 (1.1)	0.324
Moderate or severe Ovarian Hyperstimulation Syndrome	0 (0.0)	0 (0.0)		1 (0.07)	0 (0.0)	1.000*	0 (0.0)	0 (0.0)		3 (0.2)	0 (0.0)	0.038*

* Fisher's exact test

**Figure 1:**
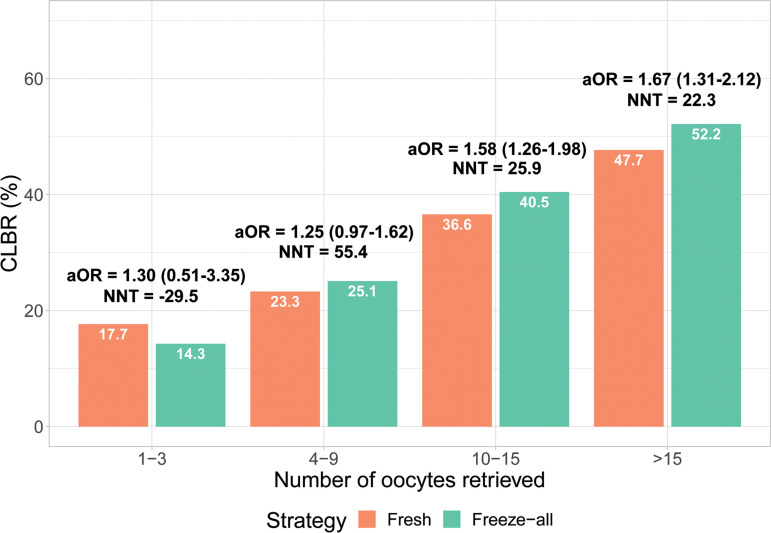
Cumulative live birth rate (CLBR) and adjusted odds ratios in groups of ovarian response of fresh and freeze-all strategy.

Multivariable logistic regression was performed to control for potential confounders when the impact of fresh and freeze-all policies on CLBR was analysed. The details of the coefficients, the aOR with a 95% confidence interval (95% CI), and the p-values of all variables can be found in Supplementary Table 1. There was no difference between freeze-all and fresh patients in Group 1 (aOR = 1.3 (0.51-3.35), *p*=0.584) and Group 2 (aOR = 1.25 (0.97-1.62), *p*=0.083). In Group 3, a significant difference was found for aOR = 1.58 (1.26-1.98), favouring the freeze-all strategy (*p*<0.001). Similarly, the freeze-all strategy was shown to substantially improve CLBR for patients in Group 4, with an aOR = 1.67 (1.31-2.12), *p*<0.001 (Figure 1).

**Supplementary Figure S1:**
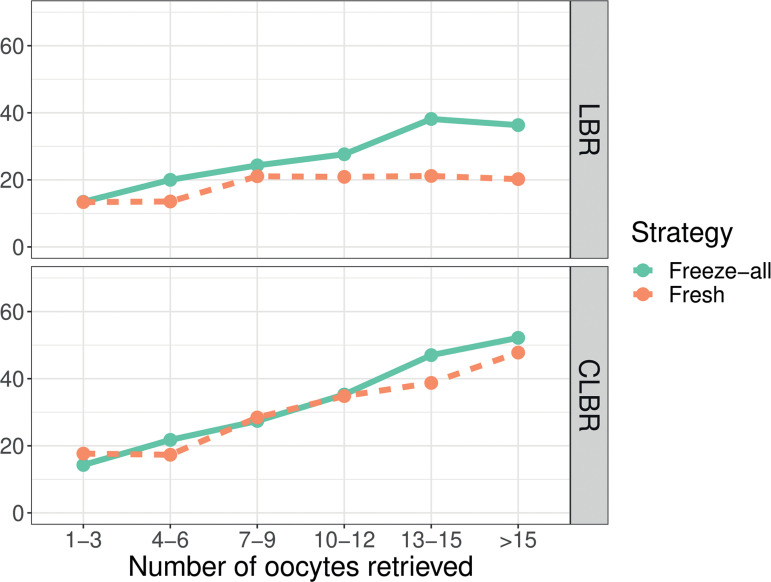
Live birth rate (LBR) and cumulative birth rate (CLBR) according to the number of oocytes retrieved.

We also performed a subgroup analysis, dividing the patients in the subgroups of 3 oocytes retrieved, in an attempt to find to most adequate number of retrieved oocytes above which implementing the freeze-all strategy would be of benefit. In this subgroup analyses (Supplemental Figure 1), the CLBR of the freeze-all and fresh groups were relatively equivalent and followed a similar trend from 1-3 oocytes to 10-12 oocytes. The CLBR of fresh patients was significantly lower than that of the freeze-all patients when the number of oocytes retrieved was 13 oocytes and above (Supplemental data).

## DISCUSSION

To our knowledge, this is the largest single centre study comparing the cumulative clinical outcomes of the fresh and freeze-all strategies based on patient's ovarian response to COS. The results of this study suggest that implementation of the freeze-all strategy, concerning clinical outcomes, might be individualized based on ovarian response, as not all groups of patients present improved CLBR per cycle with the freeze-all strategy compared with fresh ET. With the improvements in cryopreservation protocols, eFET has changed the way we currently perform IVF treatments, and the freeze-all strategy has being adopted worldwide indiscriminately, aiming to improve IVF outcomes (Nagy *et al*., 2020). Yet, despite the significant universal shift towards eFET, it is unclear if its generalized use may benefit the overall population subjected to IVF treatments (Roque *et al*., 2018a; 2019a;b).

The first randomized clinical trials (RCT) focusing on clinical outcomes dates from 2011, when Shapiro *et al*. compared the clinical outcomes of the freeze-all cycle to fresh ET in hyper (Shapiro *et al*., 2011a) and normal responders (Shapiro *et al*., 2011b), and found improvements in clinical and ongoing pregnancy rates when performing a freeze-all cycle instead of a fresh ET. Roque *et al*. (2013) published a meta-analysis evaluating the freeze-all strategy, and concluded that the freeze-all was associated with improved ongoing pregnancy rates (Relative Risk [RR] = 1.32; 95%CI 1.10-1.59; *p*=0.003) when compared with fresh cycles. However, this conclusion was based on only three RCTs evaluating a total of 633 IVF cycles. Moreover, after the publication of this meta-analysis, one of the studies was retracted from the literature due to methodological flaws, and after the removal of this study, there were no differences in ongoing pregnancy rates when comparing freeze-all with fresh cycles (RR = 1.26; 95%CI 1.00-1.58; *p*=0.05) (Roque *et al*., 2017b). However, with the publication of other studies, it became clear that the use of the freeze-all strategy for unselected groups of patients undergoing IVF treatment presented no additional benefits over fresh ET (Roque & Esteves, 2020). Recent RCTs have reported mixed results in terms of reproductive outcomes when comparing fresh ET with freeze-all in specific populations, such as patients with polycystic ovarian syndrome (PCOS) (Chen *et al*., 2016), in normo-ovulatory women with cleavage stage ET (Shi *et al*., 2018) and blastocyst ET (Wei *et al*., 2019), in women without PCOS (Vuong *et al*., 2018), and in patients undergoing preimplantation genetic testing for aneuploidy (PGT-A) (Coates *et al*., 2017). There are no RCTs available using poor responder patients. Although observational studies have not demonstrated a benefit in terms of ongoing pregnancy with the freeze-all cycle in this specific population (Roque *et al*., 2018a), there is a rationale for using this strategy with these poor prognosis patients concerning embryo pooling strategies to improve the number of available embryos and improve clinical outcomes (Blockeel *et al*., 2019).

The most comprehensive meta-analysis comparing freeze-all cycles to fresh ET, which included 5,379 from RCTs, found an overall 7% increase in LBR with the eFET strategy. However, the CLBRs were similar when both strategies in the overall population were compared. A subgroup analysis indicated that eFET was advantageous for hyper-responders, but not for normal responders (Roque *et al*., 2019a;b). In general, the studies evaluating normal responders included patients with a range of 4-15 oocytes retrieved. However, recent studies have shown that this range is not the most appropriate for classifying a normal responder, as the CLBR may vary significantly within this range of retrieved oocytes. Thus, it is more plausible to classify the ovarian response as poor (1-3 oocytes), suboptimal (4-9 oocytes), normal (10-15 oocytes), and high responders (>15 oocytes) (Polyzos & Sunkara, 2015). This classification is thought to provide a better prediction of CLBR, consequently supplying the best tailored treatment for IVF patients. Our study is the first to compare the freeze-all strategy to fresh ET into the aforementioned subgroups. Evaluating the freeze-all strategy in all of these subgroups from poor to hyper-responders is important, as all the RCTs available for evaluating the LBR following the freeze-all strategy were performed in normal and high responders with a minimum mean number of retrieved oocytes of 12 (Chen *et al*., 2016; Shapiro *et al*., 2011a;b; Shi *et al*., 2018; Vuong *et al*., 2018; Wei *et al*., 2019; Coates *et al*., 2017; Ferraretti *et al*., 1999; Aghahosseini *et al*., 2017; Aflatoonian *et al*., 2018).

In the present study, improved clinical outcomes were observed when evaluating the first ET and the CLBR for freeze-all cycles, not only in hyper-responders (>15 oocytes retrieved) but also in normal responders (10-15 oocytes retrieved). There was no benefit for performing the freeze-all strategy in suboptimal and poor responders. These findings are in accordance with most of the recent data (Roque *et al*., 2018a; Li *et al*., 2019; Bosdou *et al*., 2019; Acharya *et al*., 2018), as although there are many potential advantages to performing a freeze-all cycle over a fresh ET, it is not designed for all IVF patients (Roque *et al*., 2018b; 2019a;b). Our findings are also in accordance with studies that correlate COS with endometrium histological advancement (Ubaldi *et al*., 1997; Kolibianakis *et al*., 2002). The supra-physiologic hormonal levels that occur during a COS may be associated with modifications in the peri-implantation endometrium, which may be related to a decrease in pregnancy rates in fresh ET compared with FET. These modifications are related to an endometrial advancement that can be observed during a histological evaluation during a fresh cycle, and when this advancement occurs over 3 days, no pregnancies are achieved (Ubaldi *et al*., 1997; Kolibianakis *et al*., 2002). However, these data cannot be extrapolated to all patients subjected to COS, as the mean number of retrieved oocytes in these studies was >15, and the patients presenting no pregnancy when the endometrial advancement occurred over 3 days were those with supra-physiologic progesterone levels (*p*≥1.1 ng/ml) on the trigger day (Ubaldi *et al*., 1997; Kolibianakis *et al*., 2002). In addition, the studies that identified changes in gene expression profiles in the endometrium of patients subjected to COS that suggested ovarian hyperstimulation and high progesterone levels on the day of final oocyte maturation might be detrimental to implantation due to altered genes that are crucial for endometrium-embryo interaction were performed in oocyte donors who achieved a hyper-response to the treatment and also presented high estradiol levels (Horcajadas *et al*., 2005; Labarta *et al*., 2011). Thus, the aforementioned are related to altered endometrial patterns after COS in patients presenting a hyper-response, and are not evidence that the COS may impact the embryo-endometrium interaction in patients with poor or suboptimal response.

One of the strong points of our study is that the primary outcome was CLBR, which provides more meaningful information and a better understanding of the real efficacy of an IVF treatment. After adjusting for potential confounders, the CLBRs of the two methods were higher in groups 3 and 4 but comparable in groups 1 and 2. The increase in serum estradiol and progesterone levels on the trigger day in groups 3 and 4 might have resulted in a less receptive endometrium in fresh transfer. Our finding are not in agreement with Li *et al*. (2019) who recently compared the CLBR of the fresh and the freeze-all strategies in different subgroups of patients. They found that the freeze-all strategy resulted in a CLBR similar to fresh transfer among high responders (>15 oocytes retrieved), and was associated with a reduced likelihood of a live birth in suboptimal (1-9 oocytes) and normal (10-15 oocytes) responders. However, this study was a population-based retrospective cohort study with little information available on clinic protocols for the freeze-all policy, including intention-to-treat, embryo quality, and cryopreservation technique. Moreover, they only evaluated three subgroups of patients with different ranges of oocytes in each subgroup (Li *et al*., 2019), unlike the subgroups used in the present study.

A major limitation of our study is its retrospective design, which may be a subject of bias. In Group 4 (>15 oocytes) the ovarian reserve parameters, i.e. AMH and AFC, and the number of retrieved oocytes were significantly higher in the freeze-all group. This can be explained by the fact that we only included in the study patients who performed fresh ET after an hCG trigger, to avoid potential bias in the results for fresh cycles after an GnRH agonist trigger and fresh ET. Thus, the patients with higher ovarian response were triggered with a GnRH agonist to decrease the risk of OHSS when performing the freeze-all strategy. Moreover, a multivariable logistic regression was performed to control potential confounders, such as the ovarian reserve parameters and ovarian response to treatment, when evaluating the primary outcome (CLBR), adjusting the outcomes for these potential confounders. In the present study, we were not able to evaluate the cost-effectiveness of the freeze-all strategy. Observational studies have shown that the freeze-all strategy may be cost-effective when compared with fresh ET (Roque *et al*., 2015; Papaleo *et al*., 2017). However, more robust data is needed to establish the cost-effectiveness of the strategy.

In conclusion, the implementation of the freeze-all strategy should be individualized, as although there are many potential advantages to performing a freeze-all cycle over a fresh ET, it is not ideal for all IVF patients. Based on the present data, it seems reasonable to implement this strategy to improve the CLBR per cycle in patients presenting a hyper or a normal response to COS. Indiscriminate use of the freeze-all strategy may be associated with increased costs, laboratory workflow, and time to live birth.
